# Lack of association between the *APLNR* variant rs9943582 with ischemic stroke in the Chinese Han GeneID population

**DOI:** 10.18632/oncotarget.22588

**Published:** 2017-11-21

**Authors:** Pengyun Wang, Chuchu Wang, Sisi Li, Binbin Wang, Liang Xiong, Xin Tu, Qing K. Wang, Cheng-Qi Xu

**Affiliations:** ^1^ Department of Clinical Laboratory, Liyuan Hospital, Tongji Medical College, Huazhong University of Science and Technology, Wuhan, P. R. China; ^2^ Key Laboratory of Molecular Biophysics of the Ministry of Education, Cardio-X Institute, College of Life Science and Technology and Human Genome Research Center, Huazhong University of Science and Technology, Wuhan, P.R. China; ^3^ Zhengzhou University, School of Life Sciences, Zhengzhou, P.R. China; ^4^ National Research Institute for Family Planning, Beijing, P.R. China; ^5^ Center for Cardiovascular Genetics, Department of Molecular Cardiology, Lerner Research Institute, Department of Cardiovascular Medicine, Cleveland Clinic, Cleveland, Ohio, USA; ^6^ Department of Molecular Medicine, Department of Genetics and Genome Science, Case Western Reserve University, Cleveland, Ohio, USA

**Keywords:** atherosclerosis, ischemic stroke, association studies, APLNR, Rs9943582

## Abstract

Stroke is one of the most common causes of death worldwide. Genetic risk factors have been found to play important roles in the pathology of ischemic stroke. In a previous genome-wide association study, a functional variant (rs9943582, –154G/A) in the 5’ flanking region of the apelin receptor gene (*APLNR*) was shown to be significantly associated with stroke in the Japanese population. However, the association required validation in other ethnicities. To validate the genetic relationship between *APLNR* and ischemic stroke in the Chinese Han population, we genotyped rs9943582 in a case–control population containing 1,158 ischemic stroke patients and 1,265 common controls enrolled from the GeneID database, and performed a genetic association study. We detected no allelic or genotypic associations between rs9943582 and ischemic stroke in the Chinese Han GeneID population, although the study population provided sufficient statistical power. This finding indicates that the association between the *APLNR* variant and ischemic stroke or atherosclerosis may need further validation.

## INTRODUCTION

Stroke is one of the commonest causes of death worldwide [1]. In China, 1.5–2 million new cases of stroke occur annually, accounting for around 20% of all deaths [2–5]. Ischemic stroke is the main type of stroke, which is responsible for about 87% of stroke cases [6]. It is usually caused by a thrombus or embolus blocking blood flow to the brain, and atherosclerosis of the small or large arteries in the brain is the major cause for the formation of thrombi [7].

Epidemiological studies have indicated that genetic and environmental risk factors and their interactions play important roles in the pathology of ischemic stroke. Large-scale genome-wide association studies (GWAS) have identified loci conferring risk to ischemic stroke, including variants in 11q12.1 (*APLNR*), 12p13 (*NINJ2*), 12q24 (*ALDH2*), 7p21 (*HDAC9*), 9p21 (*ANRIL*), 4q25 (*PITX2*), 16q22 (*ZFHX3*), 9q34 (*ABO*), and 1p13.2 (*TSPAN2*) [8–14]. Candidate gene association studies also identified variants that confer risk to ischemic stroke in several susceptibility genes including *ALOX5AP*, *VKORC1, NOS3*, *PCSK9*, *PDE4D*, and *SGK1* [15–20].

In a previous GWAS conducted by Hata *et al*., a functional variant in the 5′ flanking region (rs9943582, –154G/A) of the apelin receptor gene (*AGTRL1* or *APLNR*) was found to be significantly associated with risk of stroke in the Japanese population. The apelin receptor is a G-protein-coupled receptor, which was identified as the receptor for the adipokine apelin [21–22]. In a mouse model, *APLNR* was found to be associated with atherosclerosis [23].

To validate the genetic relationship between the *APLNR* variant and ischemic stroke in the Chinese Han population, we evaluated the contribution of rs9943582 to the genetic susceptibility of ischemic stroke in the Chinese GeneID population including 1,158 ischemic stroke patients and 1,265 controls.

## RESULTS

### Characteristics of study subjects

The case group of 1,158 ischemic stroke patients had an average age of 60.6 ± 8.4 years, females accounted for 41%, 55% of the subjects had hypertension, and 13.5% had type 2 diabetes mellitus. In controls, the average age of the 1,265 subjects was 61.7 ± 10.2 years, 38.4% were females, 37.6% had hypertension, and 9.4% had type 2 diabetes. The mean age of subjects in the control group was significantly higher than those in the case group (*P* < 0.001) (Table 1), while the proportion of subjects with hypertension and type 2 diabetes was significantly higher in the case group than the control group. We also observed that serum total cholesterol levels and low density lipoprotein cholesterol levels (LDL-C) were significantly higher in cases than controls group, while the levels of high density lipoprotein cholesterol (HDL-C) were significantly lower in cases than controls. There was no significant difference in serum triglyceride content between the two groups (Table 1).

**Table 1:** Basic characteristics of the ischemic stroke case control popolation

Characteristic	Cases (*n* = 1158)	Controls (*n* = 1265)	*P*
Age (years)^*^	60.6 ± 8.4	61.7 ± 10.2	<0.001
Gender, female, *n* (%)	475 (41.0%)	486 (38.4%)	>0.05
Hypertension, *n*, (%)	637 (55%)	476 (37.6%)	<0.001
Diabetes, *n*, (%)	158 (13.5%)	119 (9.4%)	<0.01
Total Cholesterol (mmol/L)	4.67 ± 1.17	4.26 ± 1.12	<0.001
Triglyceride (mmol/L)	1.82 ± 0.65	1.79 ± 0.71	>0.05
HDL-C (mmol/L)	1.11 ± 0.33	1.20 ± 0.36	<0.001
LDL-C (mmol/L)	2.66 ± 1.06	2.45 ± 1.04	<0.001
Smoker *n* (%)	361 (31.7%)	345 (27.3%)	<0.001

Data are shown as mean +/– standard deviation (SD) for quantitative variables and *n* (%) for qualitative variables.

HDL-c: high density lipoprotein cholesterol levels; LDL-c, low density lipoprotein cholesterol levels.

^*^Age at the first diagnosis of the disease.

### No significant association between *APLNR* variant rs9943582 with ischemic stroke in the Chinese Han GeneID population

Under the population parameter setting of the effect size or odds ratio (OR) of 1.3 for stroke [14], and the minor allele frequency of 0.252 for rs9943582 (according to 1000 Genome CHB datasets), our case–control population can provide a statistical power of 98% to detect an association between rs9943582 and ischemic stroke with a type I error of 0.05. Therefore, our GeneID samples were sufficiently large to test the association between rs9943582 and ischemic stroke, and the possibility of obtaining false positives or negatives was limited.

We genotyped rs9943582 in cases and controls, and compared the frequency of alleles in each group (Table 2). The rs9943582 A allele was observed at a frequency of 26.3% in 1,158 ischemic stroke patients, compared with 25.6% in 1,265 controls. Statistical analysis indicated that there was no significant difference in allelic frequency between cases and controls: the observed *P* value (*P-obs*) was 0.57, and the OR was 1.04 (95% confidence interval (CI): 0.91–1.18) (Table 2). After adjusting for potential confounders such as age, gender, hypertension, diabetes mellitus, smoking status, and lipid concentrations (total cholesterol, triglycerides, HDL-C, and LDL-C), the association remained insignificant (OR = 0.99 with an adjusted *P* (*P-adj*) of 0.86) (Table 2).

**Table 2:** Analysis of allelic association of rs9943582 with ischemic stroke

Cohort (*n*, case/control)	Number of Genotype			Freq _A (case/control)	Observed^*^		Adjust^†^	
		Case	Control		*P-obs*	OR (95% CI)	*P-adj*	OR (95% CI)
Entire population (1158/1265)	AA	97	92	0.263/0.256	0.57	1.04 (0.91–1.18)	0.86	0.99 (0.87–1.13)
	AG	416	464					
	GG	645	709					
Male (683/779)	AA	57	61	0.276/0.258	0.28	1.10 (0.93–1.29)	0.69	0.97 (0.81–1.15)
	AG	263	278					
	GG	363	440					
Female (475/486)	AA	40	30	0.245/0.253	0.69	0.96 (0.78–1.18)	0.41	1.00 (0.88–1.36)
	AG	153	187					
	GG	282	269					
Non-Hypertension (521/789)	AA	38	66	0.256/0.245	0.50	1.06 (0.89–1.27)	0.50	0.94 (0.78–1.13)
	AG	191	254					
	GG	292	469					
Hypertension (637/476)	AA	59	26	0.269/0.275	0.75	0.97 (0.80–1.17)	0.24	1.13 (0.92–1.38)
	AG	225	210					
	GG	353	240					

Freq _A: Frequency of A allele.

^*^Uncorrected *P* value and odds ratio (OR) using Chi-square tests with Pearson's 2 × 2.

^†^Adjusted *P* value by multivariate logistic regression analysis for potential confounders including age, gender, smoking, hypertension, diabetes mellitus and lipid concentrations (Tch, TG, HDL-c and LDL-c).

We conducted subgroup analysis of the study subjects by gender, and found that the allelic association between variant rs9943582 and ischemic stroke was not significant in either the female population (475 cases versus 486 controls, *P_obs_*= 0.69, OR = 0.96; *P_ad j_*= 0.41, OR = 1.00) or the male population (683 cases versus 779 controls, *P_obs_* = 0.28, OR = 1.10; *P_adj_* = 69, OR = 0.97) (Table 2).

We also divided the study subjects according to hypertension status, and again found that the association remained insignificant. In subjects with hypertension (637 cases versus 476 controls), the *P_obs_* was 0.75 (OR = 0.96) and the *P_adj_* was 0.24 (OR = 1.13), while the *P_obs_* was 0.50 (OR = 1.06) and the *P_adj_* was 0.40 (OR = 0.94) in the subgroup without hypertension.

Further analyses included the genotypic association under three common genetic models: additive, dominant, or recessive (Table 3). No significant genotypic association was identified between variant rs9943582 and ischemic stroke under all three genetic models. Adjusting for covariates of sex, age, hypertension, smoking history, type 2 diabetes, and lipid concentration also found no significant association (Table 3).

**Table 3:** Analysis of genotypic association of rs9943582 with ischemic stroke in Chinese Han GeneID population under different genetic models of inheritance

Cohorts	Model	P_obs^*^	*OR (95% CI)*	*P_adj*^†^	*OR (95% CI)*
**Entire cohort (1,158/1,265)**	Dominant	0.89	1.01 (0.86–1.19)	0.60	1.05 (0.89–1.24)
	Recessive	0.28	1.18 (0.88–1.59)	0.59	0.91 (0.87–1.24)
	Additive	0.54	n.a	0.87	1.01 (0.89–1.15)

^*^ Uncorrected *P* value and odds ratio (OR) using Chi-square tests with Pearson's 2 × 2.

^†^Adjusted *P* value by multivariate logistic regression analysis for potential confounders including age, gender, smoking, hypertension, diabetes mellitus and lipid concentrations (Tch, TG, HDL-c and LDL-c).

n.a.: no data.

## DISCUSSION

In the present study, we performed a case–control genetic association analysis to determine whether the *APLNR* variant rs9943582, previously identified in a GWAS as conferring risk to ischemic stroke in the Japanese population [14], was also a risk factor for ischemic stroke in the Chinese GeneID population. No significant association between rs9943582 with ischemic stroke was detected in our population of 1,158 ischemic stroke patients and 1,265 controls.

Previous functional research identified an association between the apelin receptor and apelin with atherosclerosis [23]. However, no genetic associations between rs9943582 and coronary artery disease, which shares a similar pathogenic mechanism to that of stroke, were identified in our previous study of Chinese individuals or in research by Kunihiko *et al.* into Japanese and Korean populations [24–25]. Similarly, our present case–control association analysis detected no allelic or genotypic association between rs9943582 and ischemic stroke in the Chinese Han GeneID population, although the study population provided sufficient statistical power. Our result is consistent with the study by Zhang *et al.*, which also showed a negative association between rs9943582 and the age of onset and clinical outcomes of ischemic stroke in the Chinese population [26].

In the present study, we excluded ischemic stroke patients with subarachnoid hemorrhages, embolic brain infarctions, and those shown by computed tomography (CT) or magnetic resonance imaging (MRI) data to have subcritical hemispheric lesions <1.5 cm in diameter, which are commonly classified as lacunar strokes. In fact, most of the patients in the present study had cortical or cerebellar lesions and brain stem or subcortical hemispheric infarcts >1.5 cm in diameter, which are considered to potentially be of large artery atherosclerosis origin. Therefore, our findings may represent the association between rs9943582 and ischemic stroke of large artery atherosclerosis origin, so further study may be required to determine whether it confers risk to other subtypes of ischemic stroke such as lacunar stroke, cardioembolic stroke, and infarcts of uncertain cause.

One limitation of the present study is that only one variant (rs9943582) was selected to investigate the genetic association between *APLNR* and ischemic stroke risk; therefore, our study cannot exclude the possibility that other *APLNR* variants confer risk to ischemic stroke. The association between other polymorphisms in the *APLNR* genomic region and ischemic stroke requires additional study.

In conclusion, our study indicates that the *APLNR* variant rs9943582 is not associated with ischemic stroke in the Chinese Han population, and that the association between this variant and ischemic stroke or atherosclerosis needs further validation. The findings in this study further emphasize the population heterogeneity of genetic susceptibility of ischemic stroke.

## MATERIALS AND METHODS

### Study populations

The subjects in the present case–control genetic association study were enrolled from the GeneID database, which is an ongoing study of the Chinese Han population that has collected >80,000 DNA samples and available clinical data. The GeneID database aims to identify susceptibility genes or other risk factors for cardiovascular and cerebrovascular diseases in the Chinese Han population [25, 27–35]. This study was approved by the Ethics Committee of Huazhong University of Science and Technology and conforms to the Declaration of Helsinki guidelines. Written informed consent was obtained from all study subjects.

The 1,158 ischemic stroke patients were receiving treatment for ischemic stroke at Wuhan hospitals, whereas the 1,265 controls were individuals subjected to physical examination at the same hospitals. All subjects were self-reported to be of Chinese Han origin.

The diagnosis of ischemic stroke was according to standard World Health Organization criteria [36]. The clinical diagnosis was made by at least two independent neurologists based on a medical history of stroke, stroke signs by neurological examination, and cerebral ischemia by CT or MRI images. We selected patients with ischemic stroke only, and excluded those with subarachnoid hemorrhages, embolic brain infarctions, brain tumors, and those with a relevant brain stem or subcritical hemispheric lesions <1.5 cm dimeter based on CT or MRI data. The ischemic stroke subtype classification was according to the Trial of ORG 10172 in Acute Stroke Treatment [37]. We enrolled healthy individuals as controls according to physical tests and medical history.

Other basic demographic and clinical characteristics were obtained including age, gender, hypertension, type 2 diabetes mellitus, and lipid concentrations (total cholesterol, LDL-C, HDL-C, and triglycerides). Hypertension was defined as a systolic blood pressure of ≥140 mmHg or a diastolic blood pressure of 90 mmHg. Type 2 diabetes was defined as receiving ongoing therapy for diabetes or a fasting plasma glucose level of ≥126 mg /dL after at least 8 h of fasting, or a 2 h plasma glucose level of ≥200 mg /dL during an oral glucose tolerance test.

### Single nucleotide polymorphism (SNP) genotyping

Genomic DNA was prepared from venous blood samples using the Wizard^®^ Genomic DNA Purification Kit (Promega, WI, USA).

Genotyping for SNP 9943582 was performed by SYTO-9 fluorescent dye-based high-resolution melting (HRM) analysis as previously described [37]. Briefly, the PCR reaction mixture of 25 μl contained 2.5 μl of 10 × PCR Buffer (containing 15 mM MgCl_2_), 0.4 μl of dNTPs (10 mM), 0.4 μl of each primer (10 μM), 1 μl of template DNA (25 ng/μl), 5 μmol/L of SYTO-9 fluorescent dye, 0.2 U of Taq DNA polymerase, and ddH_2_O. The forward PCR primer was 5′-ACCACTTCCTGCCTGCCCTTTA-3′ and the reverse primer was 5′-ACACCCTCCTTGCTCCCTACCA-3′. PCR conditions were: 95°C for 5 min, followed by 40 cycles of 95°C for 15 s, 55°C for 15 s, and 72°C for 15 s, with a final elongation step of 72°C for 10 min. PCR products were analyzed and the genotype of each individual was determined using the HRM program. Three DNA samples with known genotypes of CC, AC, AA were included as positive controls, and one negative control of ddH_2_O without genomic DNA was also included in each genotyping assay. The total success rate of HRM genotyping was 95.2%.

HRM genotyping results were validated by direct DNA sequence analysis of 48 randomly selected subjects. The genotypes obtained from HRM analysis were shown to be 100% concordant with those from Sanger sequencing analysis.

### Statistical analysis

Statistical analysis was performed as previously described [17, 38]. The allelic frequency in cases and controls was compared by Pearson's 2 × 2 contingency tables and chi-square tests as implemented in PLINK version 1.06. *P* values and corresponding ORs with 95% CIs were computed. Multivariate logistic regression analysis was performed using SPSS version 17.0 by adjusting for risk factors (age, sex, hypertension, smoking history, type 2 diabetes, and lipid concentrations).

The statistical power analysis was performed using a free program (Power and Simple Size Calculation, 3.0.12 version) for a case–control design (http://biostat.mc.vanderbilt.edu/wiki/Main/PowerSampleSize) [39]. The statistical power of the case–control study was calculated using specific parameters, including the minor allele frequency (0.252 for rs9943582 according to CHB 1000 Genomes data), OR (1.3 according to a previous study by Hata *et al.* [14]), the numbers of cases and controls, and the type I error of 0.05. The null hypothesis could be rejected if OR = 1 with probability (power). The program uses an uncorrected chi-squared statistical method to evaluate the null hypothesis [40].
